# Exposure to opposing temperature extremes causes comparable effects on *Cardinium* density but contrasting effects on *Cardinium*-induced cytoplasmic incompatibility

**DOI:** 10.1371/journal.ppat.1008022

**Published:** 2019-08-19

**Authors:** Matthew R. Doremus, Suzanne E. Kelly, Martha S. Hunter

**Affiliations:** 1 Entomology and Insect Science Graduate Interdisciplinary Program, University of Arizona, Tucson, Arizona, United States of America; 2 Department of Entomology and Insect Science, University of Arizona, Tucson, Arizona, United States of America; Pennsylvania State University, UNITED STATES

## Abstract

Terrestrial arthropods, including insects, commonly harbor maternally inherited intracellular symbionts that confer benefits to the host or manipulate host reproduction to favor infected female progeny. These symbionts may be especially vulnerable to thermal stress, potentially leading to destabilization of the symbiosis and imposing costs to the host. For example, increased temperatures can reduce the density of a common reproductive manipulator, *Wolbachia*, and the strength of its crossing incompatibility (cytoplasmic incompatibility, or CI) phenotype. Another manipulative symbiont, *Cardinium hertigii*, infects ~ 6–10% of Arthropods, and also can induce CI, but there is little homology between the molecular mechanisms of CI induced by *Cardinium* and *Wolbachia*. Here we investigated whether temperature disrupts the CI phenotype of *Cardinium* in a parasitic wasp host, *Encarsia suzannae*. We examined the effects of both warm (32°C day/ 29°C night) and cool (20°C day/ 17°C night) temperatures on *Cardinium* CI and found that both types of temperature stress modified aspects of this symbiosis. Warm temperatures reduced symbiont density, pupal developmental time, vertical transmission rate, and the strength of both CI modification and rescue. Cool temperatures also reduced symbiont density, however this resulted in stronger CI, likely due to cool temperatures prolonging the host pupal stage. The opposing effects of cool and warm-mediated reductions in symbiont density on the resulting CI phenotype indicates that CI strength may be independent of density in this system. Temperature stress also modified the CI phenotype only if it occurred during the pupal stage, highlighting the likely importance of this stage for CI induction in this symbiosis.

## Author summary

Insects often harbor heritable symbiotic bacteria that infect their cells and/or bodily fluids. These heritable bacteria are passed from mother to offspring and can have substantial effects on host insect biology, and include bacteria like *Cardinium* that cause mating incompatibilities between symbiont-infected and uninfected individuals. Often, the extent of these symbiont-conferred modifications correlates with the bacterial density in the host. The appearance of these phenotypes is also affected by temperature stress, which often reduces bacterial density. However, here we find that temperature-altered strength of *Cardinium*-induced mating incompatibility in a whitefly parasitoid wasp can be independent of *Cardinium* density. While heat treatment reduced the symbiont density and the phenotype, as expected, cold treatment also reduced symbiont density but increased the degree of mating incompatibility. Here, the prolonged duration of the host pupal development in the cold treatments appeared to be more important for phenotype strength. These results suggest that the connection between bacterial density and phenotype strength may not be as general as previously thought. Furthermore, the modification of this manipulative phenotype has implications for the effectiveness of the host, *Encarsia suzannae*, as a biological control agent.

## Introduction

Temperature has profound effects on the biology of ectothermic organisms as well as on the microbiota inhabiting these organisms [1–4]. The heritable symbionts of arthropods, which are primarily vertically transmitted from mother to offspring, are closely integrated with host biology [5], and may be particularly vulnerable to temperature stress [4, 6]. These symbionts rely on strategies, ranging from mutualistic to parasitic, to modify host phenotype in ways that increase the likelihood of their maternal transmission [5]. While transmission of these symbionts and their phenotypic effects are usually stable under benign conditions, both aspects may be threatened as the environment becomes less hospitable [4]. Extreme temperatures can reduce symbiont density [7, 8, 9], induce phenotypic failure [7–10], and/or reduce rates of vertical transmission, resulting in uninfected offspring [8, 9]. From the host’s perspective, the limited thermotolerance of the symbiont compared to the host is a vulnerability, since long-term associations are likely to make hosts metabolically or reproductively dependent on these symbionts, even those that originally acted as reproductive parasites [11–14]. In these cases, temperature-induced disruption of these symbioses can be disastrous for the host, reducing host fitness by rendering hosts vulnerable to natural enemies [10, 15] or causing reproductive failure [13, 16, 17].

Temperature extremes thus pose a clear challenge to heritable symbioses, including those involving one of the best-studied symbionts, *Wolbachia*, a diverse genus of widespread bacteria (Phylum Alphaproteobacteria). *Wolbachia* typically acts as a reproductive parasite of hosts using one of four major strategies, the most common of which is cytoplasmic incompatibility (CI)[18]. CI is a mating incompatibility between symbiont-infected males and uninfected females, resulting in inviable offspring. Offspring viability is restored (or “rescued”) in crosses with infected females [18]. CI increases infected female fitness at the cost of uninfected female fitness, thus driving the symbiont infection through a population [19–21]. Some *Wolbachia* strains also improve host resistance to viruses and macroparasites, including several important human pathogens, instigating the use of this symbiont as a means of controlling important human diseases, including dengue fever, zika, and chikungunya [22–24].

Multiple studies using different host species and *Wolbachia* strains have found that *Wolbachia’s* CI phenotype can be modified by temperature [7, 8, 25]. These changes to CI appear to be generally mediated by changes in *Wolbachia* density, which in turn influences CI strength [26]. Temperature stress reduces *Wolbachia* density, thereby weakening CI [8, 25]. Temperature-induced *Wolbachia* reduction also leads to partial failure of the rescue phenotype and reduced efficacy of vertical transmission [8, 27]. From these studies, it seems that temperature stress in some habitats could limit the ability of some *Wolbachia* strains to maintain high levels of infection or spread through novel populations, limiting both the natural spread of this symbiont as well as efforts to use this symbiont in disease vector control [8].

While there is well over three decades of research studying the effects of temperature on *Wolbachia* symbioses [28–30], we know very little about how temperature effects a widespread and unrelated bacterium, *Cardinium hertigii* (Phylum Bacteroidetes; [31, 32]), that also induces CI in its hosts [33–37]. A CI-inducing *Cardinium* strain infects the parasitoid wasp, *Encarsia suzannae* (Hymenoptera: Aphelinidae, = *Encarsia pergandiella*; [33, 38]). Genome and transcriptome comparisons between this CI-*Cardinium* strain from *E*. *suzannae*, *c*Eper1, and CI-*Wolbachia* strains found little homology between the two bacterial genomes, indicating that CI arose independently in these distantly related symbionts [39, 40]. Despite the two bacteria using different molecular mechanisms to induce CI, the resulting phenotype is strikingly similar; both CI mechanisms ultimately kill embryos by inhibiting proper chromosome pairing in early mitotic cycles, leading to chromatin bridging between daughter cells, eventually triggering widespread aneuploidy and embryonic mortality [41, 42].

Although *Cardinium* symbioses present an alternative study system for the study of CI, research concerning the impact of environmental factors upon this manipulative symbiont is limited to a handful of studies investigating *Cardinium* presence or absence at various temperatures. One such study found that *Cardinium* prevalence was reduced in natural populations of *Culicoides* biting midges (Diptera: Ceratopogidae) occupying arid regions, offering evidence for a destabilizing effect on the symbiosis attributed to extreme temperature fluctuations in those regions [43]. More recently, an analysis of global *Cardinium* infection frequencies found increased *Cardinium* prevalence in insect host populations occupying warmer regions (mean temperature of ~25°C) compared to cooler ones [44]. Other studies using laboratory cultures of the spider mite, *Metaseiulus occidentalis*, found that mite cultures held at 34°C for >1 year were either uninfected with *Cardinium* or had a reduced amount of the symbiont compared to cultures maintained at room temperature [45, 46]. Together, these studies provide some evidence for a general destabilizing effect of extreme temperatures on *Cardinium*-host symbioses; yet we still do not know if temperature modifies other aspects of *Cardinium* symbioses, particularly the CI phenotype.

**Fig 1:**
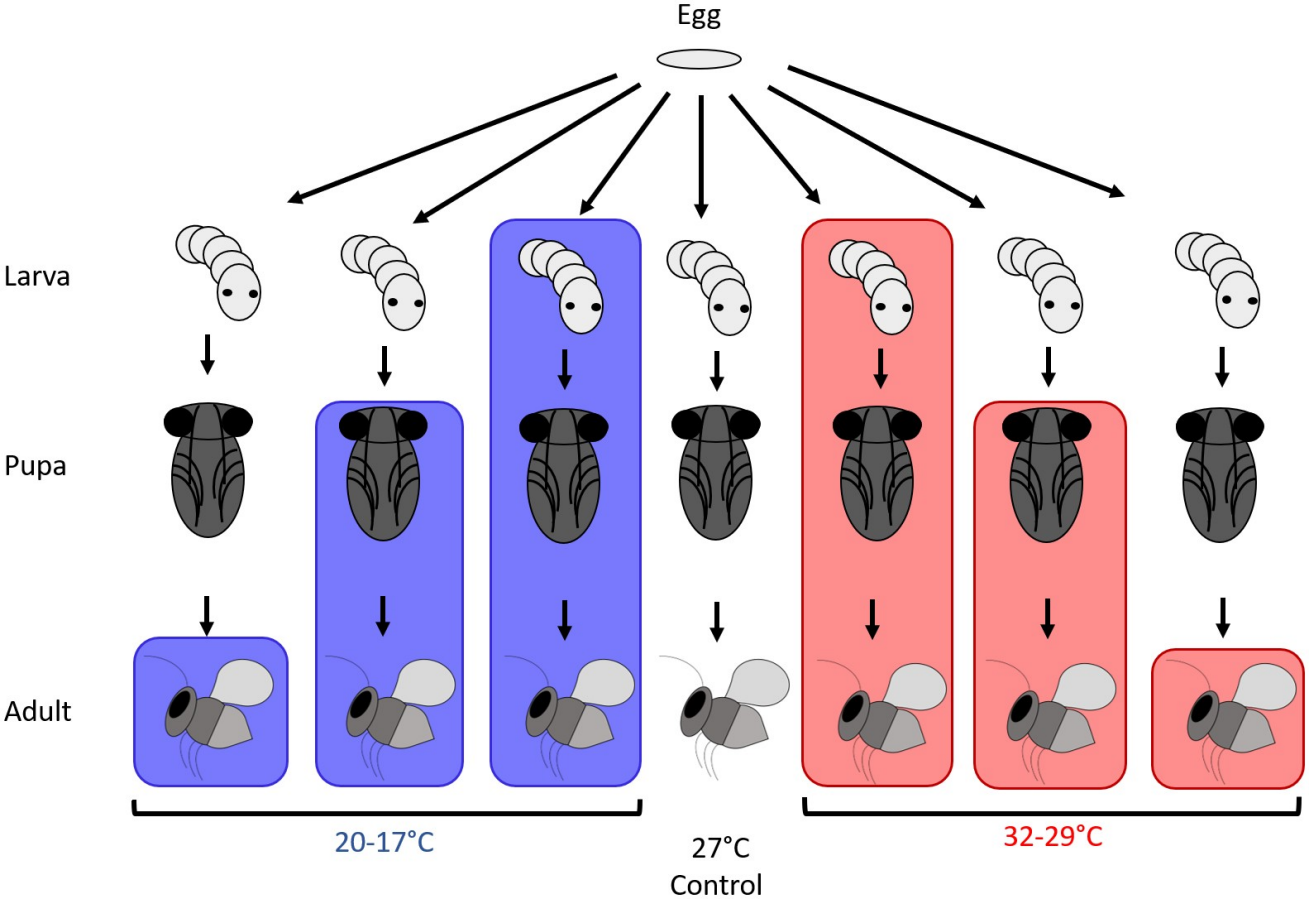
Experimental design for testing the effects of prolonged temperature exposure on *Cardinium’s* CI phenotype. Color of bars denotes temperature range experienced at that life stage (no bar = 27°C, red = 32–29°C, blue = 20–17°C). This experimental design was modified slightly for temperature shock and vertical transmission assays. In shock assays, temperature shocks did not extend to multiple life stages. Only the control and larval treatments were performed in the vertical transmission experiment.

Here we examined how temperature stress modifies this manipulative symbiosis. We tested the effects of prolonged exposure, including one or more host stages, to either high or low temperatures on both *Cardinium* CI modification (in males) and rescue (in females; Fig 1). We next tested whether observed temperature-induced changes to *Cardinium’s* CI modification machinery and rescue capabilities were due to changes in symbiont density. We also tested the effect of temperature stress on the duration of host pupal development, a host factor that appeared to correlate with *Cardinium’s* CI strength in a previous study [47]. We next used temperature shock treatments during specific life stages to test whether temperature’s influence on CI was life-stage dependent. Finally, we tested the effect of temperature stress on vertical transmission rates of this heritable symbiont.

## Results

### Warm and cool temperatures modify CI strength of *Cardinium*

**Fig 2:**
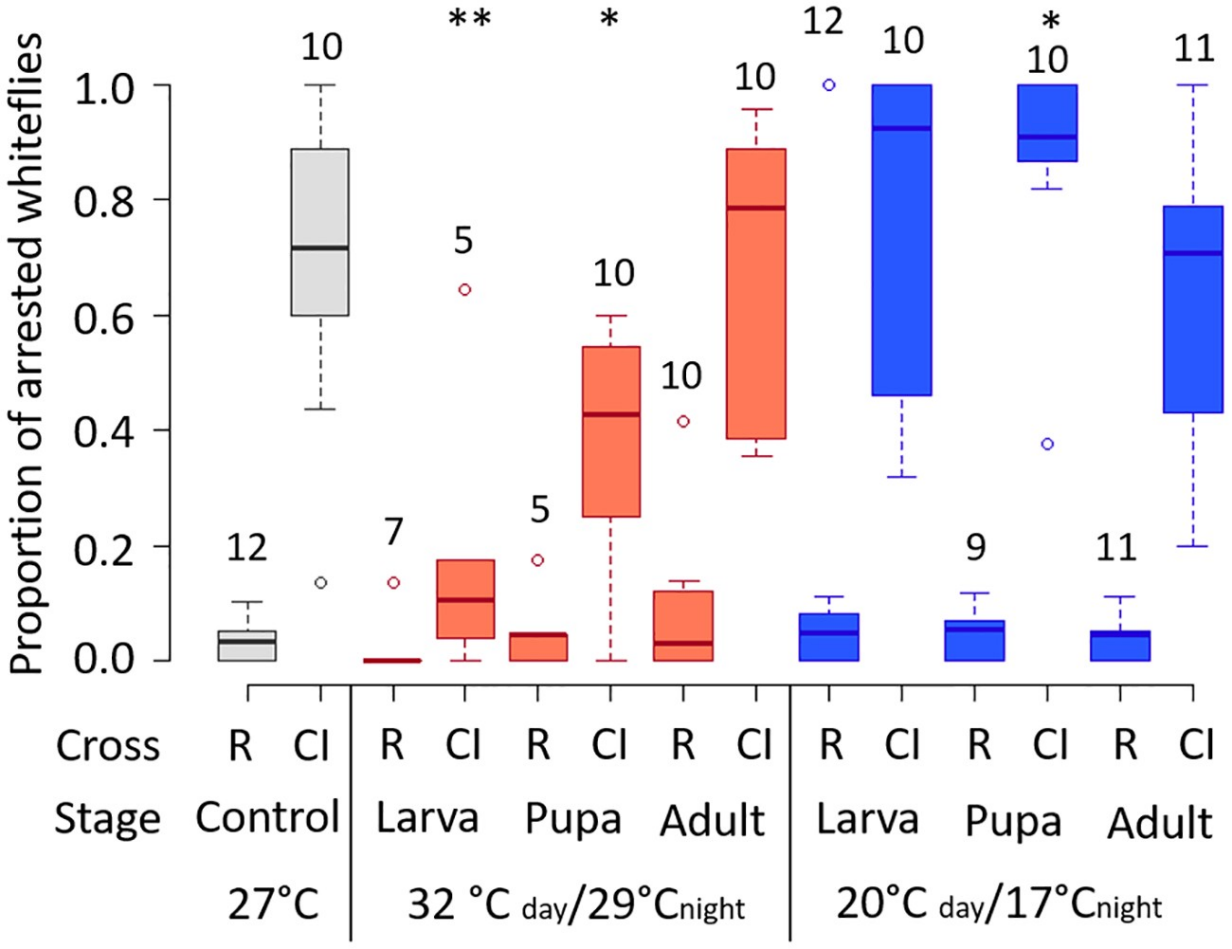
Proportion of arrested whiteflies (proxy for CI-mortality) generated from crosses involving male wasps exposed to different temperatures. Color of bars denotes temperature range (grey = 27°C, red = 32–29°C, blue = 20–17°C). Treatments are also separated by the stage at which the temperature exposure began. R = rescue cross, C+ female x C+ male. CI = CI cross, C- female x C+ male. Asterisks denote significant differences compared to crosses of the same type performed at 27°C. Numbers above whisker plots denote replicate number. N = 5–12 males, with a mean offspring per male = 17.7.

Similar to previous studies of this system, we found that at the constant temperature of 27°C, the CI cross resulted in ~70% offspring mortality (Fig 2; [33, 47–49]). We also found that prolonged exposure of male hosts to either warm (32°C day/29°C night; 16:8 h) or cool (20°C day/17°C night; 16:8 h) temperatures caused dramatically different levels of CI-induced offspring mortality compared to males reared at 27°C (Fig 2). Prolonged exposure to warm temperatures, beginning either when the host was a larva or pupa, resulted in a significant decrease in CI-induced offspring mortality (Fig 2; S1 Table; Logistic regression *F*_1,32_^larva^ = 7.15, *p* = 0.01; *F*_1,31_^pupa^ = 7.49, *p* = 0.01). Interestingly, exposure to cool temperatures beginning in the pupal stage had an opposite effect on CI strength and resulted in an increase in CI-induced mortality (*F*_1,38_ = 9.98, *p* = 0.003). In this treatment, the CI phenotype became not only stronger but also less variable than CI typical of this *Cardinium* strain (Fig 2). However, we found that neither warm nor cool temperatures affected CI strength when the exposure period only included the adult stage (Fig 2; *F*_1,37_^cool-adult^ = 0.04, *p* = 0.85; *F*_1,30_^warm-adult^ = 0.85, *p* = 0.36). We found no effect of temperature on offspring viability in control (“N”) crosses, in which no CI-induced mortality is expected, showing that changes in offspring mortality in warm and cool treatments are not due to a general effect on male viability (Fig 2; Logistic regression *F*_1,40_^cold-larva^ = 0.26, *p* = 0.62; *F*_1,39_^cold-pupa^ = 2.31, *p* = 0.14; *F*_1,38_^cold-adult^ = 0.08, *p* = 0.77; *F*_1,31_^warm-larva^ = 0.43, *p* = 0.52; *F*_1,30_^warm-pupa^ = 0.0002, *p* = 0.99; *F*_1,29_^warm-adult^ = 2.56, *p* = 0.12).

### Warm and cool temperatures modify *Cardinium*’s ability to rescue CI

**Fig 3:**
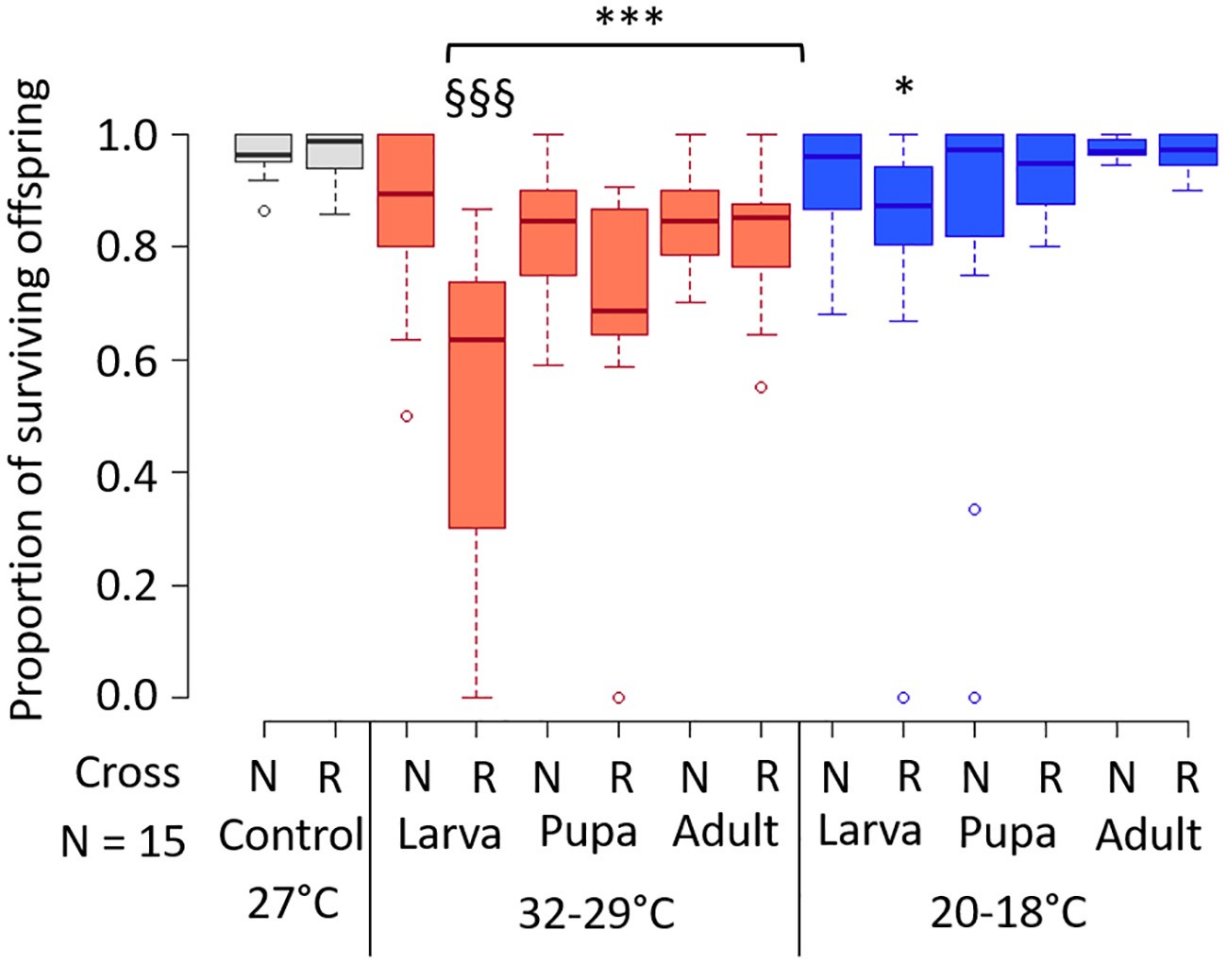
Proportion of surviving offspring (developed pupae) generated from crosses involving female wasps exposed to different temperatures. Color of bars denotes temperature range (grey = 27°C, red = 32–29°C, blue = 20–17°C). Treatments are also separated by the stage at which temperature exposure began. N = control cross: C+ female x C- male. R = rescue cross: C+ female x C+ male. Asterisks denote significant differences compared to respective crosses at 27C. § denotes significant differences to the N cross within that temperature treatment. N = 15 females for each treatment, with the mean number of offspring per female = 9.25.

When we tested the effectiveness of the ability of *Cardinium* in females to rescue *Cardinium*-modified sperm, the 27°C control treatment showed comparable offspring survival between the rescue cross (“R”; infected mother x infected father) and the control cross (“N”; infected mother x uninfected father), indicating that *c*Eper1 reliably rescues CI when mating individuals bear the same *Cardinium* strain (Fig 3; [33, 47]). We found that exposure of females to warm temperatures (32°C day/29°C night; 16:8 h) beginning during the pupal and adult stages decreased offspring survival in both R and N crosses compared to those same crosses at 27°C (Fig 3, S1 Table; Logistic regression *F*_1,54_^pupa-N^ = 11.78, *p* = 0.001; *F*_1,53_^adult-N^ = 18.26, *p* = <0.0001; *F*_1,53_^pupa-R^ = 19.02, *p* = <0.0001; *F*_1,52_^adult-R^ = 15.74, *p* = 0.0002). The heat-exposed control (N) crosses showed lower offspring survival rates than the 27°C controls, which suggests that heat reduced the viability of the experimental females independent of the CI phenotype. To account for this general reduction in viability, we subsequently compared offspring survival between the rescue (R) cross and its corresponding control (N) cross for each heat treatment. We then found an additional significant reduction in offspring survival in the R cross compared with its corresponding N cross when females were exposed to heat starting in the larval stage (Fig 3; *F*_1,28_ = 20.13, *p* = 0.0001). These results suggest a partial failure of the rescue phenotype with heat, although pupal and adult exposure to heat did not significantly influence rescue beyond the general reduction in viability (*F*_1,28_^pupa^ = 2.85, *p* = 0.1; *F*_1,25_^adult^ = 0.11, *p* = 0.74). Cool temperatures (20°C day/17°C night; 16:8 h) did not decrease offspring survival in N crosses, suggesting no effect of cold on the viability of females (*F*_1,52_^larva^ = 2.53, *p* = 0.12; *F*_1,51_^pupa^ = 0.89, *p* = 0.35; *F*_1,50_^adult^ = 0.54, *p* = 0.47). For this reason, we compared cold R rescue crosses to the R cross of females kept at the 27°C control temperature (Fig 3). Like the heat treatment, the longest cold exposure resulted in reduced offspring survival in R crosses, indicative of partial rescue failure (Fig 3; F_1,56_ = 10.65, *p* = 0.002), but neither exposure at pupal or adult stages reduced rescue significantly (*F*_1,55_^pupa^ = 3.21, *p* = 0.08; *F*_1,54_^adult^ = 0.25, *p* = 0.62).

### Warm and cool temperatures reduce *Cardinium* density in male and female hosts

**Fig 4:**
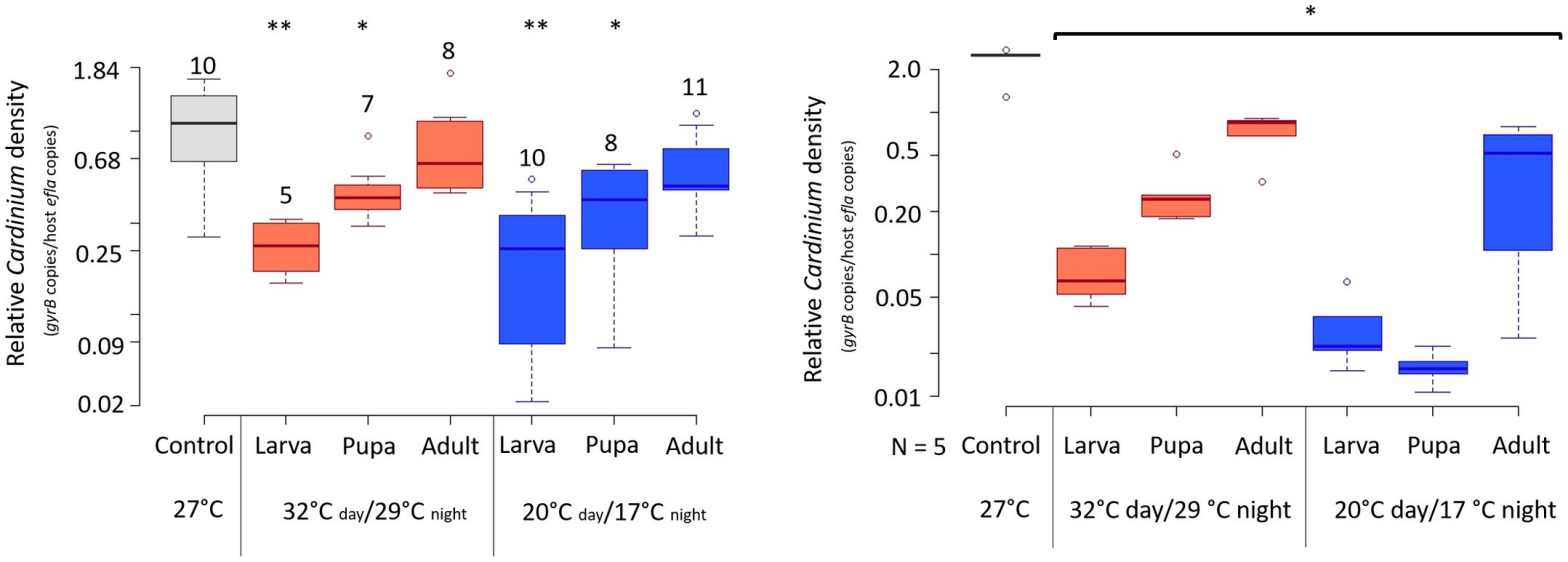
Density of *Cardinium* infection in adult a) male *E*. *suzannae* and b) female *E*. *suzannae*. Densities were measured by estimating the amount of single copy *Cardinium gyrB* genes relative to single copy host *ef1-alpa* genes with qPCR. N = 5–10 for males (denoted by numbers above whisker plots) and N = 5 for females. Each sample was run with three technical replicates. Color of bars denotes temperature range (grey = 27°C, red = 32–29°C, blue = 20–17°C). Treatments are also separated by the stage at which temperature exposure began. Asterisks denote significant differences compared to the symbiont density at 27°C.

We tested the effect of temperature stress on *Cardinium* density in male and female hosts. We found that *Cardinium* reached variable densities in adult males at the control rearing temperature of 27°C, with densities ranging from 0.25 *Cardinium*: host cells to about 2:1 *Cardinium*:host cell (Fig 4a). Both warm and cool temperatures reduced *Cardinium* density in the male larval and pupal treatments (Fig 4a, S2 Table; Mann-Whitney U-test *p*^warm-larva^ = 0.009; *p*^warm-pupa^ = 0.04; *p*^cool-larva^ = 0.004; *p*^cool-pupa^ = 0.04), although we found no significant changes to *Cardinium* density in either of the male adult treatments, which also did not show evidence of temperature-modified CI strength (Figs 2 and 4a). While warm and cool treatments both suppressed *Cardinium* density in male hosts, these temperature treatments showed contrasting effects on CI strength. Furthermore, the *Cardinium* density of individuals across treatments did not correlate with CI strength (Spearmen’s *rho* = 0.05, *p* = 0.72).

In female wasps reared at 27°C, *Cardinium* density was less variable than in males, with females harboring about 2.5 *Cardinium*: 1 host cell (Fig 4b). Temperature treatments all significantly reduced *Cardinium* density in females relative to those kept at 27°C, regardless of temperature range or exposure period (Fig 4b, S3 Table; Mann-Whitney U-test *p* = 0.012 for all comparisons to control).

### Temperature exposure during pupal stage modifies *E*. *suzannae* pupal developmental time

**Fig 5:**
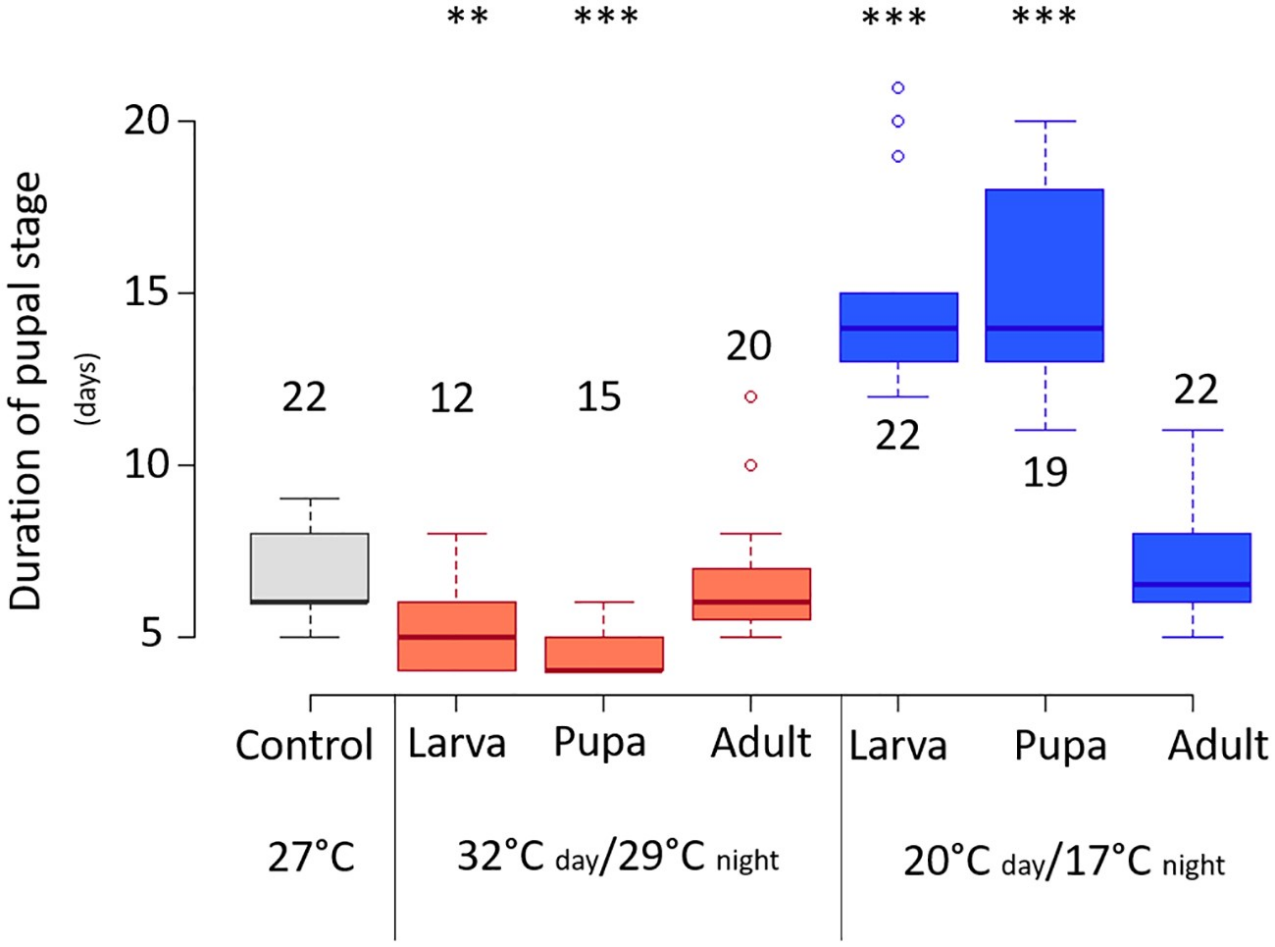
Duration of male *E*. *suzannae* pupal stage (in days) at different temperatures. Color of bars denotes temperature range (grey = 27°C, red = 32–29°C, blue = 20–17°C). Treatments are also separated by the stage at which temperature exposure began. Asterisks denote significant differences compared to the duration of the pupal stage at 27°C. Numbers above plots denotes replicates. N = 12–22 male wasps.

**Fig 6:**
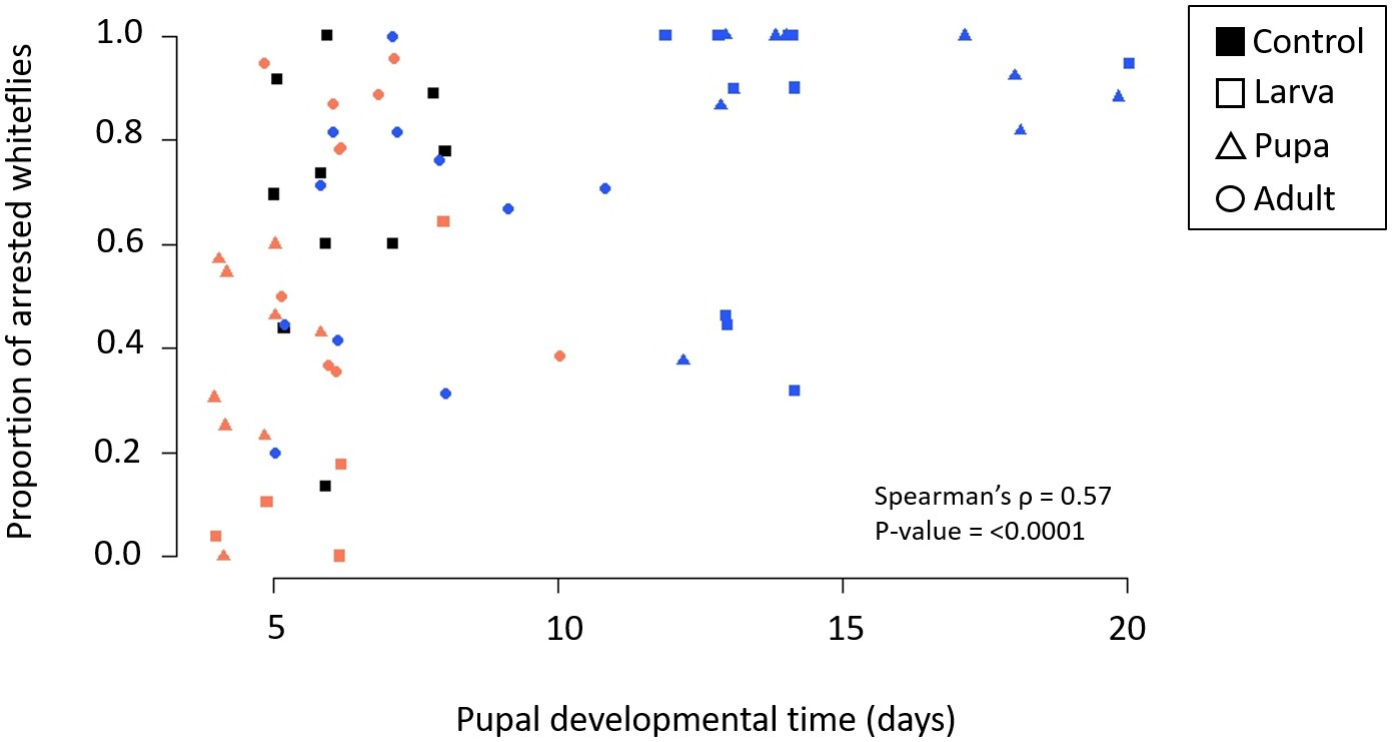
Correlation between CI strength (as denoted by the proportion of arrested whiteflies) and the duration of the pupal stage of male *E*. *suzannae*. Points refer to development time and corresponding offspring mortality for individual males used in CI crosses. The color of points denotes temperature range (black = 27°C, red = 32–29°C, blue = 20–17°C). Treatments are also separated by the stage at which temperature exposure began (□ = Larva, Δ = Pupa, O = Adult).

Because cool temperatures simultaneously reduced *Cardinium* density yet resulted in significantly stronger CI, we looked for another factor that could be responsible for the cold-modified CI phenotype. We found that cool temperatures significantly prolonged the duration of the pupal stage from ~6 days at 27°C to ~14 days (Fig 5, S4 Table; Mann-Whitney U-test *p* = <0.0001). Additionally, we found that warm temperatures significantly reduced pupal developmental time (from ~6 days to ~ 4 days) when those exposures began in the larval or pupal stage (Fig 5, S4 Table; *p* = <0.01). The adult treatments underwent their temperature exposure after the pupal stage, and we found no difference in pupal duration between adult-exposed wasps and control wasps kept at 27°C (Fig 5). As in [47], we also found that the duration of the pupal stage positively correlated with CI strength (Fig 6; Spearman’s rho = 0.57, *p* = <0.0001).

### Heat-shock during the male pupal, but not adult, stage weakens CI

**Fig 7:**
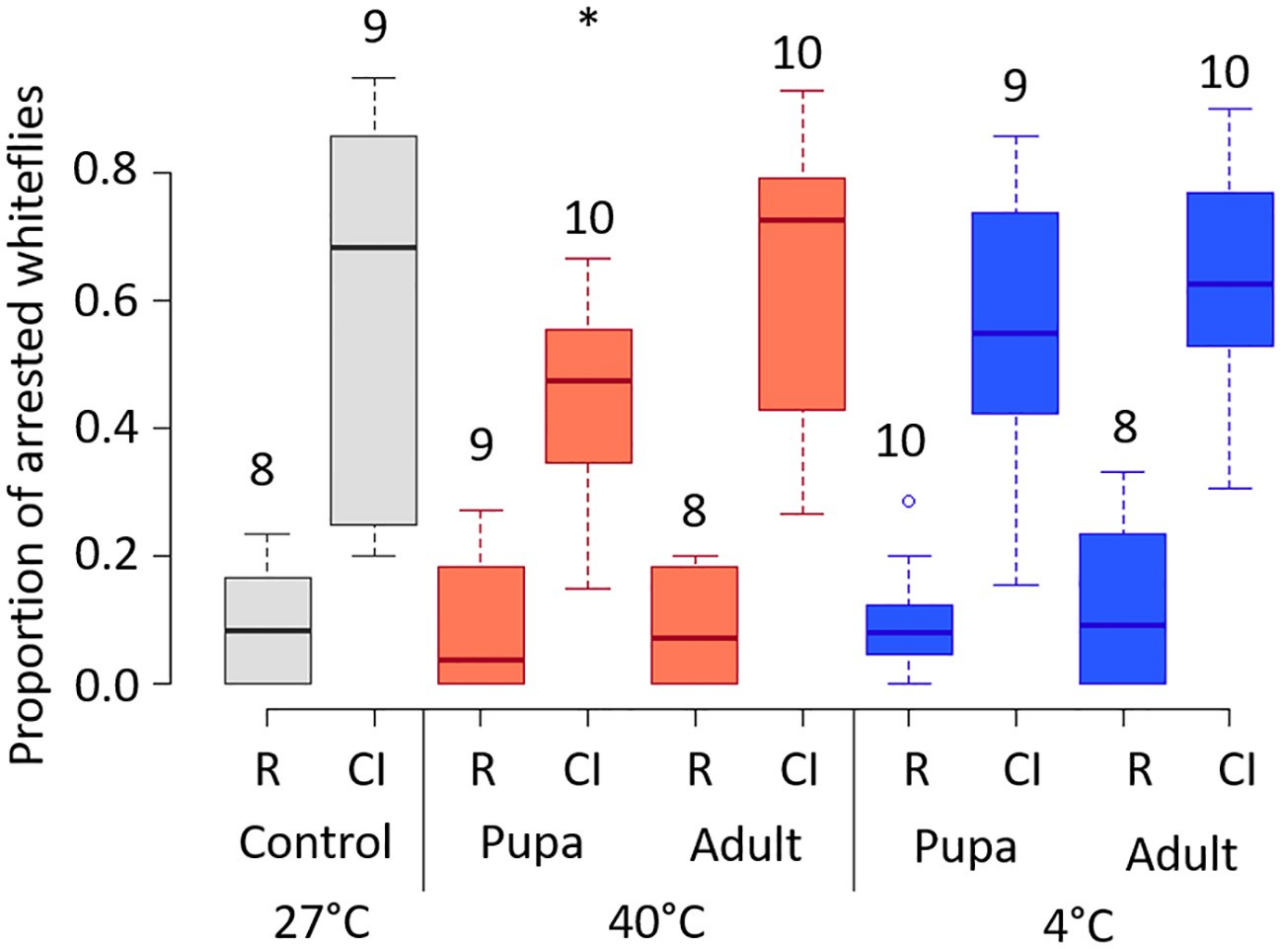
Proportion of arrested whiteflies (proxy for CI-mortality) generated from crosses involving male wasps exposed to different temperature shocks. Color of bars denotes temperature treatment (grey = 27°C, red = heat shock at 40°C, blue = cold shock at 4°C). Treatments are also separated by the stage at stage temperature exposure began. R = rescue cross, C(+) female x C(+) male. CI = CI cross, C(-) female x C(+) male. Asterisks denote significant differences compared to respective crosses at 27°C. Numbers represent replicates for each cross. N = 8–10, with the mean offspring per male = 15.3.

We designed a second experiment using temperature-shocks of equal duration to test whether temperature effects on *Cardinium*’s CI were dependent on host life stage. We found that heat shock (40°C for 2 hours) during the pupal, but not the adult stage, reduced offspring mortality in CI crosses (Fig 7, S1 Table; *F*_1,24_^pupa^ = 5.48 *p* = 0.03; *F*_1,23_^adult^ = 0.04, *p* = 0.84). Unlike our previous experiment (Fig 2), we found no difference in CI-induced mortality between the control and either the pupal or adult cold-shock (4°C for 2 hours) treatments (Fig 7, S1 Table; *F*_1,25_^pupa^ = 1.76, *p* = 0.2; *F*_1,24_^adult^ = 0.23, *p* = 0.64). Perhaps notably, cold-shock during the pupal stage did not prolong pupal development (S1 Fig). While *Cardinium* density was slightly reduced in males that experienced shocks during the pupal stage, this difference was not significantly different compared to control densities (S2 Fig).

### High temperatures reduce vertical transmission efficiency

Prolonged exposure to high and low temperatures resulted in a reduction of the ability of *Cardinium* to rescue its CI (Fig 3) and a reduction of the density in female hosts (Fig 4b). This rescue failure could have resulted from vertical transmission failure, which would generate uninfected eggs susceptible to CI. To test this hypothesis, we exposed female hosts to one of three temperature treatments that started during their larval stage: a warm (32°C day/29°C night; 16:8 h), cool (20°C day/17°C night; 16:8 h), and intermediate control (27°C) treatment, and then examined their offspring for *Cardinium* infection. We found that vertical transmission was perfect (100% of offspring were infected) at the 27°C control temperature and the cool temperature treatment (Table 1). However, heat treated wasps showed a significant reduction in the number of infected offspring, which translated to a 90% transmission rate (Table 1; Exact binomial test *p* = <0.0001).

**Table 1:** Vertical transmission rates of *c*Eper1 at various temperature ranges. Proportion of infected offspring are presented along with 95% Confidence Intervals from Exact binomial tests.

Temperature	Infected progeny/Total (proportion)	95% Confidence Intervals
27°C	114/114 (1.0)	(0.968, 1.0)
20–17°C	118/118 (1.0)	(0.969, 1.0)
32–29°C	36/40 (0.9)	(0.763, 0.972)

## Discussion

We tested the effect of temperature stress on a symbiosis between a minute parasitoid wasp, *E*. *suzannae*, and its CI-inducing heritable symbiont, *Cardinium*. Male hosts exposed to warm temperatures during the larval or pupal life stages showed faster pupal development, as well as reduced *Cardinium* density and reduced expression of the CI phenotype relative to hosts kept at a benign temperature. On the other hand, exposure to cool temperatures beginning during the pupal stage strengthened CI expression, despite also reducing *Cardinium* density. This stronger CI likely resulted from the prolonged pupal stage of cold-treated males, as cold shock treatments that did not prolong the pupal stage or modify *Cardinium* density also did not result in stronger CI. We also found that lifetime exposure to both warm and cool temperatures reduced *Cardinium* density in female hosts. Although both temperature ranges reduced *Cardinium* density in female hosts, heat exposure weakened the CI rescue phenotype more than cold. Why heat exposure caused more rescue failure is unclear, although the decreased vertical transmission rates we observed from heat-treated females to their offspring could account for additional mortality in these rescue crosses.

The symbiosis between *Cardinium* and *E*. *suzannae*, like other heritable symbioses, is vulnerable to environmental stress [4]. Specifically, warm temperatures can weaken the CI phenotype of this manipulative symbiont, with lifetime exposure to higher temperatures almost completely nullifying the phenotype. Like most symbionts, *Cardinium* infection is expected to carry a fitness cost to infected females under neutral conditions, and, like other symbionts, *Cardinium* is expected to rely on its CI phenotype to compensate for maintenance costs, allowing it to successfully invade new host populations when above the unstable equilibrium threshold [19–21, 48, 49]. With its CI phenotype weakened, the cost of harboring *Cardinium* could prevent its spread into regions experiencing prolonged bouts of warmer temperatures.

Warm temperatures also caused *Cardinium* transmission rates to drop from essentially perfect (100% of offspring infected) to a 90% infection rate. Such a decrease, coupled with modification and rescue failure, may be enough to destabilize the *Cardinium* symbiosis and cause a drop in *Cardinium* frequency in an infected population. A similar scenario occurs with *Wolbachia* that infect *Trichogramma* wasps, in which the *Wolbachia* falls in frequency during warm periods and re-invades populations in cooler months [50]. However, given that symbiont strains can show substantial variation in their susceptibility to temperature [8, 10], we caution against applying our results concerning *c*Eper1 (the *Cardinium* strain used in this study) to other strains of *Cardinium*. If a strain of *Cardinium* is thermally sensitive, like *c*Eper1, it may have dynamic infection frequencies that vary seasonally, although this should be investigated further using population-level studies and seasonal field collections.

Our study highlights the importance of the male pupal stage for this *Cardinium* strain’s CI modification step, which has mechanistic implications for *Cardinium* CI modification. Temperature exposure only affected CI strength if it occurred throughout the pupal stage, suggesting that this symbiont is actively modifying male sperm as the testes develop during the pupal stage. Furthermore, *E*. *suzannae* adult males emerge with more than enough mature sperm for ~14 successive matings in a laboratory setting (MRD, personal observation). During spermatogenesis the cytological contents, including any heritable symbionts, are dumped, leaving mature sperm bacteria-free [51, 52]. Given that male *E*. *suzannae* emerge with most of the sperm they will use in their lifetime, and that these sperm likely don’t contain *Cardinium*, we suspect that the majority of *Cardinium*’s active modification of male chromatin occurs in the pupal stage. Indeed, adult males of many parasitoid species emerge with a majority of their lifetime sperm, and the pupal stage is potentially of prime importance for the modification step of CI symbionts in these hosts [7, 53]. Future gene expression studies on *Encarsia*–*Cardinium* symbioses should focus on the pupal stage to catch expression of all possible bacterial genes involved in CI modification.

The strength of *Wolbachia* CI is generally dependent on its within-host density [8, 21, 26, 50, 52]. Reduction of *Wolbachia* density, whether by temperature or some other means, results in loss or weakening of the CI phenotype [8, 25, 54]. *Wolbachia* also harbors a bacteriophage, WO, which may further modify *Wolbachia* density and CI strength in response to extreme temperatures [7]. While we found that *Cardinium* density is also reduced by warm and cold temperature stress, this density reduction did not result in consistent changes to the CI phenotype. Instead, the duration of the pupal stage of male hosts seems to have a larger impact on this symbiont’s phenotype. This can be seen most clearly in the case of cold exposure, which prolonged the pupal stage and increased CI mortality. A similar scenario occurs in *Wolbachia*-infected *Nasonia vitripennis*, a species of parasitic wasp, that also shows prolonged pupal development and stronger CI after long-term exposure to cool temperatures. However, this *Wolbachia* strain harbors the WO phage and it’s possible that increased phage densities may also be responsible for the stronger CI phenotype [7]. It’s not known whether *Cardinium* must be present within a spermatid in order to modify it or whether the CI factors employed by *Cardinium* diffuse across membranes of developing sperm, as has been shown for *Wolbachia* [55]; either case is compatible with a time-limited mechanism for modifying sperm during the pupal stage. Extension of the pupal stage may grant time for *Cardinium* to modify a greater proportion of sperm and generate stronger CI. This contrasts with what is observed in *Drosophila melanogaster*-*Wolbachia* symbioses, in which slower developing males exhibit weaker CI through an unknown mechanism that is also independent of density [56]. Time could be a limiting factor for modification steps in CI symbioses which occur in host organisms that produce most of their lifetime sperm prior to adult emergence, like many parasitic wasps [7, 53, 57], but not for hosts that continuously mature sperm, like *Drosophila* flies [56, 58].

One caveat to the apparent lack of dependence of CI strength on density in the current study is that qPCR, our method and the standard method for estimating symbiont density, does not give any indication of symbiont health. It’s possible that while the warm and cool treatments equally depressed *Cardinium* density, the heat treatment may have damaged *Cardinium* or denatured proteins and other effector molecules involved in expression of CI, while the cold treatment only repressed *Cardinium* replication. Nevertheless, while density may be a reliable indicator of phenotypic strength for some symbionts, more examples are emerging in which phenotype strength and symbiont density are decoupled, and this rule may not be as general as originally thought [7, 9, 10, 56, 59]. In the case of low-density symbionts, like the CI-*Cardinium* strains infecting *Encarsia* wasps and citrus thrips (*Pezothrips kellyanus*) [37] or some CI-*Wolbachia* strains [59], other factors may be of equal or greater importance than symbiont density. These include host factors, like developmental time in this study, or the density of extrachromosomal elements harbored by many heritable bacteria [7]. These phages or plasmids can harbor genes responsible for the symbiont-conferred phenotype [60–63], replicate independently of the host bacterium [7, 10], and may respond differently from their host bacterium to environmental stress [7, 10]. In the case of *Wolbachia*, temperature stress inversely modifies the density of Wolbachia and its WO phage, which harbors the genes required for CI expression [7, 62]. The *c*Eper1 strain of *Cardinium* does not have a phage but does harbor a plasmid [39]. The role of the plasmid in *Cardinium* symbioses and temperature stress is not yet clear.

With a rapidly changing climate, the susceptibility of heritable symbionts to temperature stress confers similar vulnerability to the insect hosts that often depend on them, including hosts of economic or medical importance. Here, we show that a symbiosis involving *Cardinium* and its wasp host is modified by temperature. Whether this has cascading effects upon the host population remains to be seen. However, *Encarsia* spp. are used as widespread biological control agents for agricultural pests like whiteflies and armored scales [64–68]. Given the potential for temperature to disrupt these symbioses, further investigations into how environmentally susceptible *Cardinium*-*Encarsia* symbioses impact wasp and whitefly populations are warranted.

## Materials and methods

### Insect and symbiont cultures

*Encarsia suzannae* (= *E*. *pergandiella* [38]) is a species of autoparasitic wasp (Hymenoptera: Aphelinidae). Like all Hymenoptera, these wasps are haplodiploid; females develop from fertilized, diploid eggs and males develop from haploid, unfertilized eggs. In autoparasitic wasps, females lay diploid eggs within whitefly nymphs, where they develop as solitary primary parasitoids. Male eggs are laid within developing aphelinid whitefly parasitoids enclosed within the remnant whitefly cuticle, and male *E*. *suzannae* develop obligately as hyperparasitoids [69]. Laboratory cultures of female *E*. *suzannae* were maintained on *Bemisia tabaci* (sweet potato whitefly) grown on *Vigna unguiculata* (cowpea); male wasps were maintained by exposing pupae of a second parasitoid species, *Eretmocerus emiratus*, to virgin *E*. *suzannae* females. Wasp cultures were kept at 27°C and 16 D: 8 N photoperiod at ambient humidity unless otherwise noted.

The *E*. *suzannae* culture of this study was collected from the Rio Grande Valley of Texas, USA [33]. This wasp naturally harbors a CI-inducing strain of *Cardinium hertigii*, *c*Eper1 [33, 39, 40]. Additionally, an uninfected wasp culture was established by feeding adults antibiotic-spiked honey, allowing for mating crosses between uninfected C(-) and *c*Eper1 infected C(+) individuals [33]. Uninfected individuals used in this study were from a culture that had been antibiotically-cured at least 10 generations previously. The *Er*. *emiratus* used for generating male *E*. *suzannae* were not infected with *Cardinium*.

### Testing the effect of temperature stress on *Cardinium*-induced CI

Male C(+) wasps were generated by placing several (~5–7) virgin C(+) *E*. *suzannae* in a 35mm Petri dish with 25 *Er*. *emiratus* pupae on moist filter paper along with a small amount of honey. We observed females parasitize for 3 hours, removing parasitized *Er*. *emiratus* with a fine paint brush to limit superparasitism (multiple eggs oviposited in a single host). Parasitized *Er*. *emiratus* (i.e. developing male *E*. *suzannae*) were placed into individual 1.2 ml vials. Then, using an experimental design modified from [7], we tested the effect of temperature stress on *Cardinium’s* ability to induce CI in crosses between C(+) males and C(-) females by exposing these developing males to a variety of temperature treatments (Fig 1). One subset of males was kept at 27°C in a Percival incubator throughout development to serve as a control group. Other males were kept in Percival incubators set to either a “warm” or “cool” temperature range of 32°C day/ 29°C night or 20°C day/ 17°C night, respectively. We monitored internal incubator temperatures with HOBO data loggers. These ranges, particularly the “warm” temperatures, reflect the temperature extremes at which *E*. *suzannae* will tolerate constant exposure in the laboratory (MRD, personal observation). Temperature treatments were further split into three groups that began at either the larval 1^st^ instar (4-day old wasps), pupal (7-day old), or adult (upon emergence) stage [70]. All temperature treatments continued until two days post-adult emergence. This resulted in a total of seven temperature treatments that differed in their temperature range as well as the host life stages that they included (Fig 1). As *c*Eper1’s CI phenotype was previously found to positively correlate with male pupal developmental time [47], we also recorded the duration of the pupal stage for each individual male.

Two days after male adult emergence, males were mated either with virgin C(+) females for the control cross (“N”), to test for temperature-induced effects on general male viability, or with virgin C(-) females for the inviable “CI” cross to test for the effect of temperature on the strength of *Cardinium’s* CI phenotype. Mating was visually confirmed by observing male and female wasps confined in a 1.2 ml vial plugged with cotton for ~5 minutes or until mating. Mating typically occurred < 1 min after bringing the pair together, but in cases where mating had not yet occurred after 5 minutes, the female was removed, discarded, and replaced. After mating, males were stored at -80°C and females were transferred to individual arenas to parasitize whiteflies. The arenas consisted of a 35mm Petri dish with a ventilated lid, containing a cowpea leaf disc resting on 1% agar. The cowpea leaf disc bore ~50 2^nd^– 3^rd^ instar *B*. *tabaci* nymphs. Females were allowed to parasitize whiteflies in these arenas for 24hrs and the dish was then incubated at 27°C. Ten days later, we counted the number of whiteflies that either contained a developing *E*. *suzannae* pupa (resulting from a successful cross) or were developmentally arrested (i.e. parasitized by *E*. *suzannae* but the wasp failed to develop–a reliable proxy for the dead wasp embryos indicative of CI [33]). We then compared the proportion of arrested whiteflies across treatments using logistic regression with a quasibinomial distribution. We compared male pupal developmental time using the non-parametric Kruskal-Wallis test and *post hoc* multiple pairwise Mann-Whitney U Tests with Benjamini-Hochberg-corrected p-values in R v 3.3.1 [71]. We also compared male pupal developmental time and CI strength using Spearmen’s rho correlation.

### Testing the effect of temperature stress on *Cardinium*’s rescue phenotype

To test the effect of temperature stress of *Cardinium’s* ability to rescue its CI phenotype, we first generated female C(+) *E*. *suzannae* by allowing mated C+ female *E*. *suzannae* to parasitize leaf dishes with 2^nd^-3^rd^ instar whiteflies for 24hrs. Leaf discs with developing female wasps were then exposed to one of seven temperature treatments in Percival incubators as previously described. Two-days after adult emergence, experimental females were mated with either C(-) males (N; control cross) to test for temperature-induced effects on female viability, or C(+) males (R; rescue cross) that developed at 27°C to test for effects on *Cardinium’s* rescue phenotype. Here a decrease in offspring survival relative to the control would indicate a reduction in the rescue ability of *Cardinium* in the temperature treated female host. After mating observations, females were transferred to individual parasitism arenas and given 24hrs to parasitize whitefly hosts. Females were then removed and stored at -80°C. Ten days later, we counted the number of surviving wasp pupae and arrested whiteflies and analyzed rescue efficiency across temperature treatments using logistic regression with a quasibinomial distribution, as in the CI experiment.

### Quantifying *Cardinium* relative density post-temperature exposure

After the crossing experiments, we next tested whether changes in *Cardinium’s* CI modification and rescue phenotypes were due to changes in the within-host density of this symbiont. Experimental wasps (both male and female) had been stored at -80°C for DNA extraction. Extractions consisted of homogenizing a single wasp in 3 μL of 20 mg ml ^-1^ proteinase k, then adding the homogenate to 50 μL of 5–10% w/v Chelex [72]. Samples were incubated at 37°C for 1 hour with periodic vortexing, followed by incubation at 97°C for 8 min and storage at -20°C. We estimated *Cardinium* density relative to host cells by performing quantitative PCR (qPCR) using Maxima SYBR Green/ROX qPCR Master Mix (2×) (ThermoFisher Scientific) with primers for the single-copy *Cardinium gyrB* gene [47] and the host *Ef1a* gene on a Bio-Rad CFX Connect Real-Time cycler [73]. We also created standards via serial dilutions of PCR products from both primer sets. PCR products were diluted to a concentration of 1.0 ng/μL DNA, which was confirmed with a Qubit 4.0 fluorometer prior to serial dilution. Samples were run in triplicate and each qPCR plate included standards for both primer sets to correct for between-plate differences in reaction efficiency. Raw Cq values were averaged and corrected before conversion to relative density [47].

We then compared *Cardinium* density across temperature treatments for male and female wasps using the Kruskal-Wallis non-parametric test with *post hoc* multiple pairwise Mann-Whitney U Tests with Benjamini-Hochberg-corrected p-values. We also compared *Cardinium* density and CI strength using Spearmen’s rho correlation. All statistical analyses were performed in R v. 3.3.1 [71].

### Using temperature-shocks to standardize exposure period

The experiments described in the current study tested the effect of prolonged temperature stress on the *Cardinium* CI phenotype and thus included one or more host life stages and different durations of temperature exposure. To test for life stage-specific effects of temperature stress on the CI phenotype, we performed temperature “shocks” of uniform duration on male C(+) *E*. *suzannae*, during either their pupal stage or upon adult emergence. Temperature shocks consisted of a two-hour ramping step, where temperatures increased from 27°C to 40°C (heat shock) or decreased from 27°C to 4°C (cold shock), followed by a two-hour period at 40°C or 4°C, and then a two-hour ramp back to 27°C in a Percival incubator. After the temperature shock, males were maintained at 27°C until mating two days after emergence. As in the previous CI experiment, males were kept in individual 1.2 ml vials with a small amount of honey throughout development. Mating and parasitism were otherwise performed and analyzed as in the first experiments, and males were kept at -80°C for DNA extractions and *Cardinium* density estimates using qPCR.

### Testing the effects of temperature on vertical transmission efficiency

In addition to modifying symbiont-induced phenotype and/or density, temperature stress can destabilize symbiont vertical transmission [8]. Inefficient or “leaky” vertical transmission can slow the spread or cause the loss of symbionts within a population. We tested the effect of temperature stress on *Cardinium* vertical transmission by exposing C(+) female *E*. *suzannae* to one of three temperature treatments: a 27°C control, 32°Cday/29°Cnight (warm), or 20°Cday/17°Cnight (cool). Female wasps were generated as previously described and kept at 27°C until they were 4-days old (1^st^ instar larvae), when they were moved to their respective temperature treatments. Exposure continued through their development until two-days post adult emergence, when the females were mated to C(-) males, a control cross that should generate viable progeny regardless of female infection status. After mating, females were placed on arenas with whitefly nymphs and given 24hrs to parasitize hosts. These offspring were kept at 27°C until adult emergence, at which point they were frozen at -80°C. Offspring DNA was extracted and tested for the presence of *Cardinium* DNA using *Cardinium*-specific 16S rRNA primers [49]. PCRs included a positive and negative control and were run on a 1% agarose gel with SYBR Safe to visualize the product. Negative samples were re-run with the *Cardinium* primers. We next confirmed the presence of wasp DNA in negative samples using general CO1 primers [74]. Negative samples that failed to show DNA bands with the CO1 primers were discarded from the final analysis. We then tested whether the vertical transmission rates were significantly lower than 1.0 (perfect transmission) using the Exact Binomial test with 95% confidence intervals.

## Supporting information

S1 Table Logistic regression equations for comparisons of offspring mortality in temperature assays. Significance values and/or test statistics (F-value) for equation coefficients and model tests presented.
(DOCX)

S2 Table Significance values from post-hoc multiple pairwise Mann-Whitney tests with Benjamini-Hochberg corrected values comparing *Cardinium* density in male adult hosts exposed to different temperature treatments. Kruskal-Wallis χ^2^ = 29.05, df = 6, p = <0.0001.
(DOCX)

S3 Table Significance values from post-hoc multiple pairwise Mann-Whitney tests with Benjamini-Hochberg corrected values comparing *Cardinium* density in female adult hosts exposed to different temperature treatments. Kruskal-Wallis χ^2^ = 29.99, df = 6, p = <0.0001.
(DOCX)

S4 Table Significance values from post-hoc multiple pairwise Mann-Whitney tests with Benjamini-Hochberg corrected values comparing male pupal developmental time at different temperature treatments. Kruskal-Wallis χ^2^ = 43.31, df = 6, p = <0.0001.
(DOCX)

S5 Table Information on primers used in qPCR for estimating *Cardinium* density or PCR in vertical transmission studies. (DOCX)

S1 Fig Pupal developmental time for males in the heat-shock experiments. Pupal development was measured in days. Error bars show standard error. N = 17–19 for all treatments.
(TIF)

S2 Fig *Cardinium* relative density in adult wasps post-temperature shock treatments. Density was measured by estimating the amount of *Cardinium gyrB* compared to host *ef1a* genes. N = 5 for all treatments. There were no significant differences between treatments (Mann-Whitney U-tests).
(TIF)
