# Supplementary material for: Exposure to opposing temperature extremes causes comparable effects on Cardinium density but contrasting effects on Cardinium-induced cytoplasmic incompatibility
Source: PLoS Pathog. 2019 Aug 19;15(8):e1008022. doi: 10.1371/journal.ppat.1008022 (PMC6715252; doi:10.1371/journal.ppat.1008022)
Supplement: S1 Table — Significance values and/or test statistics (F-value) for equation coefficients and model tests presented. (DOCX) [file ppat.1008022.s001.docx]

| **CI experiment** | **Logistic regression equation** | **Coefficient p-values** | **LRT**  **F-value, p-value** |
| --- | --- | --- | --- |
| R cross: 27C x 20-17C | Y = -3.27 + 0.02^larva^ + 0.58^pupa^ + 0.11^adult^ | Intercept = < 0.0001  Larva = 0.946  Pupa = 0.137  Adult = 0.774 | Larva = 0.26, 0.616  Pupa = 2.31, 0.136  Adult = 0.08, 0.774 |
| CI cross: 27C x 20-17C | Y = 0.26 + 0.61^larva^ + 1.65^pupa^ - 0.09^adult^ | Intercept = 0.488  Larva = 0.299  Pupa = 0.013  Adult = 0.845 | Larva = 0.1, 0.75  Pupa = 9.98, 0.003  Adult = 0.04, 0.845 |
| R Cross: 27C x 32-29C | Y = -3.27 – 0.08^larva^ – 0.43^pupa^ – 0.89^adult^ | Intercept = < 0.0001  Larva = 0.93  Pupa = 0.541  Adult = 0.124 | Larva = 0.43, 0.52  Pupa = 0.0002, 0.988  Adult = 2.56, 0.121 |
| CI Cross: 27C x 32-29C | Y = 0.71 – 2.17^larva^ – 1.49^pupa^ – 0.45^adult^ | Intercept = 0.041  Larva = 0.005  Pupa = 0.011  Adult = 0.363 | Larva = 7.49, 0.01  Pupa = 7.15, 0.012  Adult = 0.85, 0.363 |
| **Rescue experiment** |  |  |  |
| N cross: 27C x 20-17C | Y = 3.21 - 0.56^larva^ - 0.32^pupa^ + 0.42^adult^ | Intercept = <0.0001  Larva = 0.278  Pupa = 0.601  Adult = 0.465 | Larva = 2.53, 0.118  Pupa = 0.89, 0.349  Adult = 0.541, 0.466 |
| R cross: 27C x 20-17C | Y = 3.20 - 1.09^larva^ - 0.53^pupa^ + 0.24^adult^ | Intercept = <0.0001  Larva = 0.01  Pupa = 0.23  Adult = 0.618 | Larva = 10.65, 0.002  Pupa = 3.21, 0.079  Adult = 0.25, 0.618 |
| N Cross: 27C x 32-29C | Y = 3.21 – 1.01^larva^ - 1.76^pupa^ - 1.58^adult^ | Intercept = <0.0001  Larva = 0.02  Pupa = <0.0001  Adult = 0.0001 | Larva = 0.35, 0.555  Pupa = 11.78, 0.001  Adult = 18.26, <0.0001 |
| R Cross: 27C x 32-29C | Y = 3.2 - 2.80^larva^ - 2.17^pupa^ - 1.67^adult^ | Intercept = <0.0001  Larva = <0.0001  Pupa = <0.0001  Adult = 0.0005 | Larva = 28.99, <0.0001  Pupa = 19.02, <0.0001  Adult = 15.74, 0.0002 |
| 32-29C N x R Larva | Y = -2.2 + 1.8^rescue^ | Intercept = <0.0001  Rescue = 0.0003 | Rescue = 20.13, 0.0001 |
| 32-29C N x R Pupa | Y = -1.5 + 0.46^rescue^ | Intercept = <0.0001  Rescue = 0.103 | Rescue = 2.85, 0.102 |
| 32-29C N x R Adult | Y = -1.53 – 0.10^rescue^ | Intercept = <0.0001  Rescue = 0.738 | Rescue = 0.11, 0.738 |
| **Temperature Shock** |  |  |  |
| N Cross: 27C x Cold Shock | Y = -2.36 – 0.01^pupa^ + 0.39^adult^ | Intercept = <0.0001  Pupa = 0.981  Adult = 0.523 | Pupa = 0.17, 0.687  Adult = 0.42, 0.525 |
| CI Cross: 27C x Cold Shock | Y = 0.73 – 0.70^pupa^ – 0.22^adult^ | Intercept = 0.043  Pupa = 0.178  Adult = 0.638 | Pupa = 1.76, 0.197  Adult = 0.23, 0.638 |
| N Cross: 27C x Heat Shock | Y = -2.36 – 0.33^pupa^ + 0.05^adult^ | Intercept = <0.0001  Pupa = 0.624  Adult = 0.946 | Pupa = 0.35, 0.562  Adult = 0.005, 0.946 |
| CI Cross: 27C x Heat Shock | Y = 0.73 – 1.02^pupa^ – 0.11^adult^ | Intercept = 0.043  Pupa = 0.043  Adult = 0.841 | Pupa = 5.49, 0.028  Adult = 0.04, 0.841 |
