# Supplementary material for: Exposure to opposing temperature extremes causes comparable effects on Cardinium density but contrasting effects on Cardinium-induced cytoplasmic incompatibility
Source: PLoS Pathog. 2019 Aug 19;15(8):e1008022. doi: 10.1371/journal.ppat.1008022 (PMC6715252; doi:10.1371/journal.ppat.1008022)
Supplement: S2 Table — Kruskal-Wallis χ2 = 29.05, df = 6, p = <0.0001. (DOCX) [file ppat.1008022.s002.docx]

| **Treatment** | **27C** | **Warm-Larva** | **Warm-Pupa** | **Warm-Adult** | **Cool-Larva** | **Cool-Pupa** |
| --- | --- | --- | --- | --- | --- | --- |
| **Warm-Larva** | 0.009 | - | - | - | - | - |
| **Warm-Pupa** | 0.04 | 0.03 | - | - | - | - |
| **Warm-Adult** | 0.42 | 0.008 | 0.06 | - | - | - |
| **Cool-Larva** | 0.004 | 0.91 | 0.07 | 0.007 | - | - |
| **Cool-Pupa** | 0.04 | 0.23 | 0.81 | 0.17 | 0.19 | - |
| **Cool-Adult** | 0.08 | 0.008 | 0.23 | 0.49 | 0.007 | 0.38 |
