# Supplementary material for: Exposure to opposing temperature extremes causes comparable effects on Cardinium density but contrasting effects on Cardinium-induced cytoplasmic incompatibility
Source: PLoS Pathog. 2019 Aug 19;15(8):e1008022. doi: 10.1371/journal.ppat.1008022 (PMC6715252; doi:10.1371/journal.ppat.1008022)
Supplement: S3 Table — Kruskal-Wallis χ2 = 29.99, df = 6, p = <0.0001. (DOCX) [file ppat.1008022.s003.docx]

| **Treatment** | **27C** | **Warm-Larva** | **Warm-Pupa** | **Warm-Adult** | **Cool-Larva** | **Cool-Pupa** |
| --- | --- | --- | --- | --- | --- | --- |
| **Warm-Larva** | 0.012 | - | - | - | - | - |
| **Warm-Pupa** | 0.012 | 0.012 | - | - | - | - |
| **Warm-Adult** | 0.012 | 0.012 | 0.022 | - | - | - |
| **Cool-Larva** | 0.012 | 0.039 | 0.012 | 0.012 | - | - |
| **Cool-Pupa** | 0.012 | 0.012 | 0.012 | 0.012 | 0.111 | - |
| **Cool-Adult** | 0.012 | 0.325 | 0.69 | 0.167 | 0.039 | 0.012 |
