# Supplementary material for: Exposure to opposing temperature extremes causes comparable effects on Cardinium density but contrasting effects on Cardinium-induced cytoplasmic incompatibility
Source: PLoS Pathog. 2019 Aug 19;15(8):e1008022. doi: 10.1371/journal.ppat.1008022 (PMC6715252; doi:10.1371/journal.ppat.1008022)
Supplement: S4 Table — Kruskal-Wallis χ2 = 43.31, df = 6, p = <0.0001. (DOCX) [file ppat.1008022.s004.docx]

| **Treatment** | **27C** | **Warm-Larva** | **Warm-Pupa** | **Warm-Adult** | **Cool-Larva** | **Cool-Pupa** |
| --- | --- | --- | --- | --- | --- | --- |
| **Warm-Larva** | 0.0046 | - | - | - | - | - |
| **Warm-Pupa** | <0.0001 | 0.051 | - | - | - | - |
| **Warm-Adult** | 0.595 | 0.013 | <0.0001 | - | - | - |
| **Cool-Larva** | <0.0001 | <0.0001 | <0.0001 | <0.0001 | - | - |
| **Cool-Pupa** | <0.0001 | <0.0001 | <0.0001 | <0.0001 | 0.321 | - |
| **Cool-Adult** | 0.601 | 0.0042 | <0.0001 | 0.349 | <0.0001 | <0.0001 |
