# Supplementary material for: Exposure to opposing temperature extremes causes comparable effects on Cardinium density but contrasting effects on Cardinium-induced cytoplasmic incompatibility
Source: PLoS Pathog. 2019 Aug 19;15(8):e1008022. doi: 10.1371/journal.ppat.1008022 (PMC6715252; doi:10.1371/journal.ppat.1008022)
Supplement: S5 Table — (DOCX) [file ppat.1008022.s005.docx]

| **Primer name** | **Gene (Organism)** | **qPCR?** | **Primer sequence**  **(5’ -3’)** | **Citation with PCR steps** |
| --- | --- | --- | --- | --- |
| EF_F | Ef1-alpha (*Encarsia*) | Yes | AGATGCACCACGAAGCC | Stouthamer et al. 2018 |
| EF_R | Ef1-alpha (*Encarsia*) | Yes | CCTTGGGTGGGTTGTTCTT | Stouthamer et al. 2018 |
| gyrB737F | gyrB  (*Cardinium*) | Yes | AAGTTATTGTAGCCGCTCAAG | Perlman et al. 2014 |
| gyrB911R | gyrB  (*Cardinium*) | Yes | GCAGTACCACCAGCAGAG | Perlman et al. 2014 |
| Ch441F | 16S rRNA  (*Cardinium*) | No | GTACAGGAGCAAACAATCCC | Harris et al. 2010 |
| Ch1017R | 16S rRNA  (*Cardinium*) | No | ATTTTTCAAAGTAGCAAAATA | Harris et al. 2010 |
| LCO1490 | CO1  (Mitochondrial) | No | GGTCAACAAATCATAAAGATATTGG | Folmer et al. 1994 |
| HCO2198 | CO1  (Mitochondrial) | No | TAAACTTCAGGGTGACCAAAAAATCA | Folmer et al. 1994 |
