# Supplementary figures and images for: Exposure to opposing temperature extremes causes comparable effects on Cardinium density but contrasting effects on Cardinium-induced cytoplasmic incompatibility
Source: PLoS Pathog. 2019 Aug 19;15(8):e1008022. doi: 10.1371/journal.ppat.1008022 (PMC6715252; doi:10.1371/journal.ppat.1008022)

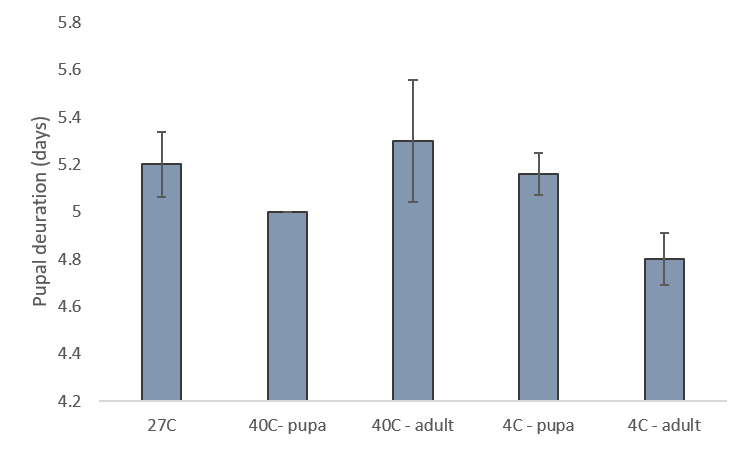

Supplement: S1 Fig — Pupal development was measured in days. Error bars show standard error. N = 17–19 for all treatments. (TIF) [file ppat.1008022.s006.tif]

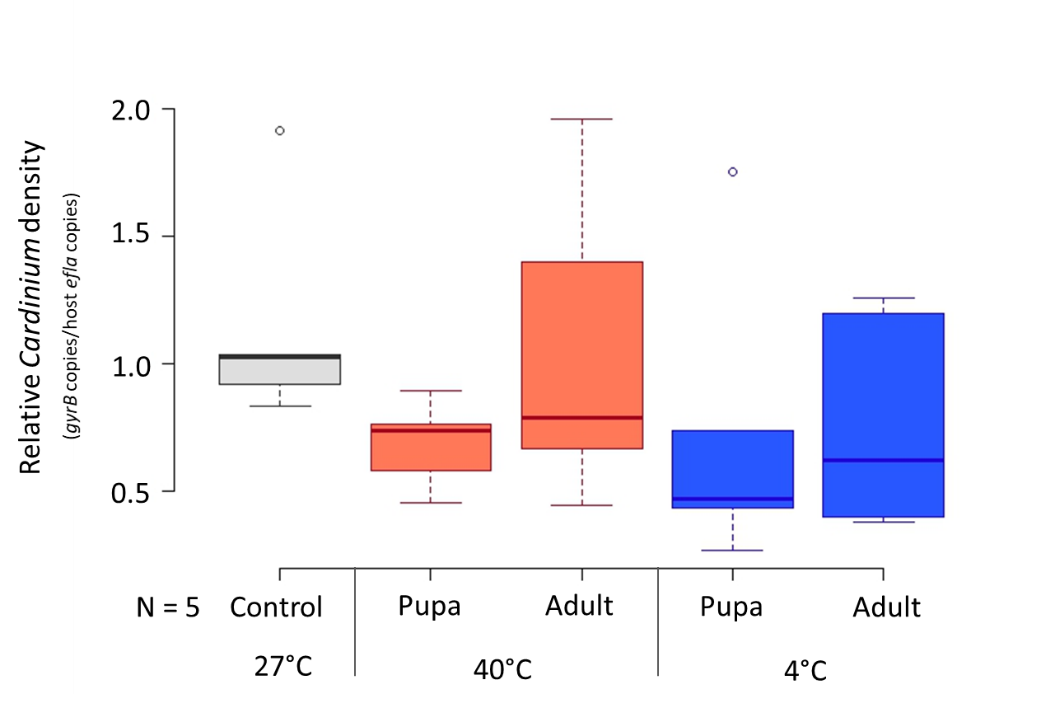

Supplement: S2 Fig — Density was measured by estimating the amount of Cardinium gyrB compared to host ef1a genes. N = 5 for all treatments. There were no significant differences between treatments (Mann-Whitney U-tests). (TIF) [file ppat.1008022.s007.tif]
